# Does smoking cause lower educational attainment and general cognitive ability? Triangulation of causal evidence using multiple study designs

**DOI:** 10.1017/S0033291720003402

**Published:** 2022-06

**Authors:** Suzanne H. Gage, Hannah M. Sallis, Glenda Lassi, Robyn E. Wootton, Claire Mokrysz, George Davey Smith, Marcus R. Munafò

**Affiliations:** 1 Department of Psychological Sciences, University of Liverpool, Liverpool, UK; 2 School of Psychological Science, University of Bristol, Bristol, UK; 3 MRC Integrative Epidemiology Unit at the University of Bristol, Bristol, UK; 4 Translational Science and Experimental Medicine, Research and Early Development, Respiratory & Immunology BioPharmaceuticals R&D, AstraZeneca, Cambridge, UK; 5 Clinical Psychopharmacology Unit, University College London, London, UK; 6 School of Population Health Sciences, Bristol Medical School, University of Bristol, Bristol, UK

**Keywords:** ALSPAC, cognition, smoking

## Abstract

**Background:**

Observational studies have found associations between smoking and both poorer cognitive ability and lower educational attainment; however, evaluating causality is challenging. We used two complementary methods to explore this.

**Methods:**

We conducted observational analyses of up to 12 004 participants in a cohort study (Study One) and Mendelian randomisation (MR) analyses using summary and cohort data (Study Two). Outcome measures were cognitive ability at age 15 and educational attainment at age 16 (Study One), and educational attainment and fluid intelligence (Study Two).

**Results:**

Study One: heaviness of smoking at age 15 was associated with lower cognitive ability at age 15 and lower educational attainment at age 16. Adjustment for potential confounders partially attenuated findings (e.g. fully adjusted cognitive ability *β* −0.736, 95% CI −1.238 to −0.233, *p* = 0.004; fully adjusted educational attainment *β* −1.254, 95% CI −1.597 to −0.911, *p* < 0.001). Study Two: MR indicated that both smoking initiation and lifetime smoking predict lower educational attainment (e.g. smoking initiation to educational attainment inverse-variance weighted MR *β* −0.197, 95% CI −0.223 to −0.171, *p* = 1.78 × 10^−49^). Educational attainment results were robust to sensitivity analyses, while analyses of general cognitive ability were less so.

**Conclusion:**

We find some evidence of a causal effect of smoking on lower educational attainment, but not cognitive ability. Triangulation of evidence across observational and MR methods is a strength, but the genetic variants associated with smoking initiation may be pleiotropic, suggesting caution in interpreting these results. The nature of this pleiotropy warrants further study.

## Introduction

Cigarette smoking is associated with numerous adverse health outcomes. Smoking prevalence has declined in high-income countries in recent years, but this decline has been strongly socially patterned, with the greatest declines in the most advantaged sections of society (Jefferis, Power, Graham, & Manor, 2004; Peretti-Watel, Seror, Constance, & Beck, 2009). As a result, smoking is now a major driver of health inequalities between advantaged and disadvantaged socioeconomic groups. There is evidence that educational attainment causally influences the likelihood of smoking initiation, and also the heaviness of smoking and likelihood of quitting among smokers (Davies, Dickson, Davey Smith, van den Berg, & Windmeijer, 2018; Gage, Bowden, Davey Smith, & Munafo, 2018; Sanderson, Davey Smith, Bowden, & Munafo, 2019; Tillmann et al., 2017). However, it is less clear whether smoking has an impact on educational attainment.

There is some evidence that young people with a history of smoking show poorer academic and occupational outcomes compared to individuals who do not smoke or desist soon after a period of experimentation (Chassin, Presson, Pitts, & Sherman, 2000; Ellickson, Tucker, & Klein, 2001; Orlando, Tucker, Ellickson, & Klein, 2004; Orpinas, Lacy, Nahapetyan, Dube, & Song, 2016). More generally, substance use in adolescence has been reported to be associated with poorer educational attainment (Fergusson, Horwood, & Ridder, 2005; Latvala et al., 2014; Maralani, 2014). However, it is possible that the observed negative association between substance use and both cognitive ability and educational attainment may result from students who experience academic failure being more likely to engage in substance use, for example in an attempt to manage resulting negative feelings (Beman, 1995; Brunswick & Messeri, 1984; Cox, Zhang, Johnson, & Bender, 2007). We assessed the association between cigarette smoking and cognitive ability and educational attainment in adolescents, hypothesizing that cigarette smoking would be associated with lower cognitive and educational outcomes.

We first used data from a large UK-based pregnancy cohort, the Avon Longitudinal Study of Parents and Children (ALSPAC), to investigate whether smoking, measured at age 15, was associated with cognitive ability, assessed at the same age, and educational attainment at age 16, before and after adjustment for a range of potential confounders. We attempted to minimise the impact of reverse causation by adjusting for pre-existing general cognitive ability (in the general cognitive ability analyses) or earlier educational attainment (in the education analyses). In an attempt to account for selection bias due to the typical attrition of large cohorts, as a sensitivity analysis, we imputed up to 100 datasets and repeated the analyses.

We next used Mendelian randomisation (MR) analyses of publicly-available summary data to confirm these findings in an independent sample. MR uses genetic variants as proxies for the exposure of interest to overcome problems of residual confounding and reverse causality (Davey Smith & Ebrahim, 2003; Davey Smith & Hemani, 2014), allowing for stronger causal inference. This use of multiple methods, each with different and ideally uncorrelated strengths, limitations, and sources of bias allows the triangulation of results to support more robust causal inference (Gage, Munafo, & Davey Smith, 2016; Lawlor, Tilling, & Davey Smith, 2016; Munafo & Davey Smith, 2018).

## Study One

### Methods

#### Data sources

The Avon Longitudinal Study of Parents and Children (ALSPAC) is a prospective, population-based pregnancy cohort study that recruited 14 541 pregnant women living in Avon, UK, with expected delivery dates between 1 April 1991 and 31 December 1992. Of these initial pregnancies, there were a total of 14 062 live births, and 13 988 children were alive at 1 year of age. The cohort has been described in detail previously (Boyd et al., 2013; Fraser et al., 2013). Ethics approval for the study was obtained from the ALSPAC Ethics and Law Committee and the Local Research Ethics Committees. Please note that the study website contains details of all the data that is available, through a fully-searchable data dictionary and variable search tool (http://www.bris.ac.uk/alspac/researchers/our-data).

Smoking heaviness was measured at age 15 via self-report questionnaire administered during attendance at a clinic session. Participants were asked ‘how many times have you smoked a cigarette in your lifetime?’ with the options never, 1–4, 5–20, 21–60, 61–100, and 101+ times.

At age 15, participants were administered the Vocabulary and Matrix Reasoning subsections of the Wechsler Abbreviated Scale of Intelligence (WASI) (Wechsler, 1999). General cognitive ability was calculated for each individual, adjusted for age. Scores were rescaled around the complete-case sample to a mean of 100 and a standard deviation of 15. General cognitive ability was also measured via the Wechsler Intelligence Scale for Children 3^rd^ Edition (Wechsler, Golombok, & Rust, 1992) at clinic at age 8.

In England, children attending state-maintained schools are educated in line with the National Curriculum, which is split into a series of ‘Key Stages’ assessed by compulsory teacher assessments of national tests (http://www.gov.uk/national-curriculum/overview). Data linkage between ALSPAC and the National Pupil Database (a central repository for pupil-level educational data in England) provided educational assessment data for participants who attended state-funded school at Key Stage 4 (age 16). Data linkage was performed by a third-party company and checked by the ALSPAC team (for further information, see http://www.bristol.ac.uk/alspac/researchers/our-data/linkage/). Educational attainment at the age of 16 was quantified using a standard capped scoring method (see http://nationalpupildatabase.wikispaces.com/KS4) in which grades achieved at General Certificate of Secondary Education (GCSE) or equivalent for their best eight subjects were converted to a numerical score (A* = 58 points … G = 16 points) and summed. Capped scores (maximum = 464) were converted to a percentage for each individual.

A number of covariates were included that may confound the association between smoking and general cognitive ability or educational attainment. These were chosen after studying previous literature and in line with Mokrysz et al. (2016). They were conceptualised as follows. Maternal and pre-birth covariates: maternal education, offspring gender, maternal depression during pregnancy, maternal alcohol, cannabis and tobacco use during pregnancy, all assessed via a maternal questionnaire during pregnancy. Childhood covariates: hyperactivity and conduct problems at age 11, assessed via the strengths and difficulties questionnaire (Goodman, 1997); depression at age 12, assessed via the moods and feelings questionnaire (Costello & Angold, 1988); psychotic experiences at age 12, assessed via the PLIKS semi-structured interview (Zammit et al., 2009); suspected truancy at age 14 assessed via maternal questionnaire. Substance use: alcohol, cannabis and other illicit drug use were measured at age 15 in the same session as the exposure.

#### Statistical analyses

Linear regression was used to assess the association between cigarette smoking at age 15 and both: (a) general cognitive ability at age 15, and (b) educational attainment at age 16, before and after adjustment for potential confounders. Consistent with the work of Mokrysz et al. (2016) we excluded individuals who had a maternal report of a head injury that resulted in unconsciousness due to the likelihood of impact on cognitive development (Azouvi, Arnould, Dromer, & Vallat-Azouvi, 2017). Furthermore, we excluded any participant who responded ‘yes’ to the question ‘have you ever used spanglers’ (a fictitious recreational drug added to the questionnaire as a quality control check). We attempted to minimise the impact of reverse causation by initially adjusting for pre-existing general cognitive ability (in the general cognitive ability analyses) or earlier educational attainment (in the education analyses) (model 2). We assessed the impact of confounding by comparing unadjusted estimates (model 1) to those adjusting for pre-birth confounders (model 3) and childhood confounders (model 4). We further adjusted for cannabis (model 5a), alcohol (5b) and illicit drugs (5c), before finally running a fully adjusted model including cannabis, alcohol and illicit drugs together (6). This approach was used to elucidate the specific impact of the covariates, given that in our previous work we have found that these variables are highly correlated with each other, and therefore that their individual impact on the strength of the association could be informative (e.g. Gage et al., 2015). All analyses were conducted in Stata version 13 (StataCorp LP, College Station, TX). The complete case sample *N* for both analyses was 2107.

#### Sensitivity analyses

In order to investigate whether selection bias due to attrition could be impacting on the complete case analysis, multiple imputations with chained equations up to the outcome sample size was carried out for each analysis. An interaction term was included to allow us to account for earlier head injury (Gage et al., 2015). We imputed up to 100 datasets, using approximately 50 auxiliary variables to predict missing values. These included further pre-birth and childhood variables, other measures of intelligence or cognition, and earlier substance use measures. The imputed sample *N* was 4954 for the general cognitive ability analysis, and 12 004 for the education analysis (as this variable was from linkage data and therefore available on many more individuals).

### Results

At age 15, 28.5% of the complete case sample of 2107 individuals reported having smoked a cigarette, and 7.5% reported smoking more than 100 times. Table 1 shows the descriptive statistics of the covariates included in the model, stratified by the heaviness of smoking. Smoking was associated with all covariates other than maternal depressive symptoms.

**Table 1.** Descriptive statistics for covariates by smoking heaviness group in the ALSPAC cohort

Smoking	Never	1–4	5–20	21–60	61–100	101+	*p*
Sample (CCA) % (*N*)	71.5 (1507)	6.7 (141)	6.6 (139)	5.1 (107)	2.7 (56)	7.4 (157)	
Female	48.6 (733)	64.5 (91)	67.6 (94)	67.3 (72)	55.4 (31)	61.2 (96)	<0.001
No higher maternal education	13.6 (205)	17.0 (24)	17.3 (24)	15.0 (16)	21.4 (12)	24.2 (38)	<0.001
Smoking during pregnancy	10.4 (156)	18.4 (26)	19.4 (27)	15.0 (16)	12.5 (7)	28.7 (45)	<0.001
Weekly alcohol use during pregnancy	12.1 (182)	22.0 (31)	21.6 (30)	11.2 (12)	21.4 (12)	15.9 (25)	0.02
Cannabis use during pregnancy	1.0 (15)	2.8 (<5)	3.6 (5)	5.6 (6)	0	2.6 (<5)	0.01
Truancy from school (age 14)	0.7 (10)	0.7 (<5)	2.2 (<5)	3.7 (<5)	1.8 (<5)	8.3 (13)	<0.001
Hyperactivity >3 (age 11)	39.8 (600)	47.5 (67)	38.1 (53)	54.2 (58)	50.0 (28)	59.9 (94)	<0.001
Conduct problems >3 (age 11)	11.0 (165)	14.9 (21)	13.7 (19)	21.5 (23)	25.0 (23)	22.3 (35)	<0.001
Psychotic-like experiences (age 12)	10.0 (150)	9.9 (14)	13.0 (18)	12.2 (13)	19.6 (11)	18.5 (29)	<0.001
Lifetime cannabis use >20 times (age 15)	1.0 (15)	2.1 (<5)	7.2 (10)	10.3 (11)	32.1 (18)	47.1 (74)	<0.001
Lifetime alcohol use >20 times (age 15)	24.6 (370)	49.7 (70)	63.3 (88)	72.0 (77)	87.5 (49)	88.5 (139)	<0.001
Illicit drug use since 15^th^ birthday	6.1 (92)	14.9 (21)	28.1 (39)	33.6 (36)	35.7 (20)	56.1 (88)	<0.001
Mean (s.d.)							
Maternal depressive symptoms	3.28 (2.23)	3.52 (2.25)	3.71 (2.26)	3.80 (2.13)	3.83 (2.93)	3.70 (2.16)	0.221
Depressive symptoms (age 12)	3.86 (1.80)	3.96 (1.76)	4.22 (1.80)	4.36 (1.76)	4.41 (1.94)	4.54 (1.77)	<0.001
General cognitive ability (age 8)	100.7 (15.3)	96.2 (14.2)	100.5 (13.6)	98.2 (15.3)	96.6 (16.1)	96.5 (13.8)	<0.001
Educational performance (age 10/11)	74.0 (12.6)	69.1 (13.3)	72.3 (12.0)	70.3 (13.8)	68.9 (14.0)	68.9 (13.0)	<0.001

CCA, complete case analysis.

#### General cognitive ability (observational analysis in ALSPAC)

Heaviness of smoking at age 15 was associated with a reduction in the general cognitive ability of 1.4 IQ points per increase in the category of smoking heaviness (95% CI −1.77 to −0.95), in an unadjusted analysis. After adjustment for earlier general cognitive ability, the magnitude of this association attenuated by over a third, although evidence of an association remained strong. Further adjustment for pre-birth and childhood confounders attenuated the association slightly further, but again strong evidence of an association remained. The addition of cannabis, alcohol or other illicit drugs slightly increased (model 5a and 5b) or decreased (model 5c) the size of the association. The fully adjusted model suggested a reduction in the general cognitive ability of 0.7 IQ points per increase in the category of smoking heaviness (e.g. between those who responded ‘never’ and those who responded ‘1–4 times’). This equates to a difference of 3.5 IQ points between never smokers and those who had smoked 101+ cigarettes at the point the questionnaire was administered (95% CI −1.24 to −0.23). The results are shown in Table 2.

**Table 2.** Linear regression analysis of smoking heaviness at age 15 and general cognitive ability at age 15, before and after adjustment for potential confounders in ALSPAC (*N* = 2107)

Model	*β* coefficient	95% CI	*p* value
1	−1.362	−1.773 to −0.950	<0.001
2	−0.882	−1.220 to −0.544	<0.001
3	−0.721	−1.059 to −0.384	<0.001
4	−0.674	−1.021 to −0.327	<0.001
5a	−0.728	−1.199 to −0.257	0.002
5b	−0.722	−1.139 to −0.304	0.001
5c	−0.618	−1.007 to −0.229	0.002
6	−0.736	−1.238 to −0.233	0.004

Model 1 – Standardised general cognitive score at age 15 by a unit increase of 5-level smoking heaviness category at age 15; Model 2 – as model 1 with additional adjustment for general cognitive ability at age 8; Model 3 – as model 2 with additional adjustment for pre-birth confounders (gender, maternal education, maternal smoking, alcohol and cannabis use during pregnancy, maternal depression); Model 4 – as model 3 with additional adjustment for childhood confounders (truancy, hyperactivity, conduct problems, psychotic-like experiences, depression); Model 5a – as model 4 with additional adjustment for cannabis use at age 15; Model 5b – as model 4 with additional adjustment for alcohol use at age 15; Model 5c – as model 4 with additional adjustment for illicit drug use at age 15; Model 6 – as model 4 with additional adjustment for cannabis, alcohol and other illicit drug use at age 15.

#### Educational attainment (observational analysis in ALSPAC)

Heaviness of smoking was associated with a 2.5 percentage point decrease in educational attainment score per increase in the category of smoking heaviness in the unadjusted analysis. This equates to a 12.5 percentage point difference between never smokers and those who had smoked 101+ cigarettes (95% CI −2.87 to −2.17). A 6 point change in score is roughly equivalent to one grade change in one GCSE examination (Lessof et al., 2018). As for associations with general cognitive ability, the effect size attenuated substantially after adjustment for earlier educational attainment. Adjustment for childhood covariates and substance use further attenuated the associations, although strong evidence of an association remained. The fully adjusted model (model 6) indicated a 1.3 percentage point decrease in education score associated with each increase in smoking heaviness category (95% CI −1.60 to −0.91). These results are shown in Table 3.

**Table 3.** Linear regression of smoking heaviness at age 15 and educational attainment at age 16, before and after adjustment for potential confounders in ALSPAC (*N* = 2017)

Model	*β* coefficient	95% CI	*p* value
1	−2.517	−2.868 to −2.166	<0.001
2	−1.725	−1.969 to −1.482	<0.001
3	−1.749	−1.986 to −1.512	<0.001
4	−1.561	−1.800 to −1.321	<0.001
5a	−1.322	−1.645 to −0.999	<0.001
5b	−1.464	−1.751 to −1.176	<0.001
5c	−1.396	−1.664 to −1.129	<0.001
6	−1.254	−1.597 to −0.911	<0.001

Model 1 – Educational performance score (as % of total possible) at age 16 by a unit increase of 5-level smoking heaviness category at age 15; Model 2 – as model 1 with additional adjustment for educational attainment at age 11; Model 3 – as model 2 with additional adjustment for pre-birth confounders (gender, maternal education, maternal smoking, alcohol and cannabis use during pregnancy, maternal depression); Model 4 – as model 3 with additional adjustment for childhood confounders (truancy, hyperactivity, conduct problems, psychotic-like experiences, depression); Model 5a – as model 4 with additional adjustment for cannabis use at age 15; Model 5b – as model 4 with additional adjustment for alcohol use at age 15; Model 5c – as model 4 with additional adjustment for illicit drug use at age 15 Model 6 – as model 4 with additional adjustment for cannabis, alcohol and other illicit drug use at age 15.

#### Multiple imputations

In the 100 imputed datasets, unadjusted associations between cigarette use and cognitive ability were somewhat smaller in magnitude than those seen in the complete case analysis (effect estimate −1.17, 95% CI −1.43 to −0.92). Adjustment for pre-birth and childhood confounders led to further attenuation. There was a small attenuation after further adjustment for cannabis, alcohol or other illicit drug use. Although effect sizes were smaller, the confidence intervals were consistent with those from the complete case analysis.

Conversely, the associations between cigarette use and educational attainment were larger in the imputed analyses than the complete case analysis (effect estimate −4.69, 95% CI −5.05 to −4.34). The pattern of attenuation was similar to that seen in the complete case analyses, although for the most part confidence intervals did not overlap with the complete case analyses. These results are shown in online Supplementary Tables S1 and S2.

### Discussion

Our results indicate that smoking cigarettes at age 15 was associated with reduced cognitive ability (i.e. vocabulary and reasoning) as measured at age 15 by the WASI, and with poorer educational attainment (i.e. grades in key stage 4 national tests) at age 16. In an attempt to minimise the possibility that our results were due to cognitive ability and educational attainment making individuals more likely to smoke, we adjusted our analyses for pre-existing general cognitive ability and earlier educational attainment. This attenuated the observed associations, although strong evidence of an association remained. However, it is important to note that this pattern of attenuation is also consistent with a null causal effect and measurement error being present (Fewell, Davey Smith, & Sterne, 2007).

Associations also remained after adjustment for substance use, although we did observe some attenuation, consistent with evidence that substance use is associated with poor cognitive performance and educational attainment (Cox et al., 2007). Multiple imputation of missing data allowed us to predict missing variables and, consequently, to include a larger number of ALSPAC subjects in the analysis. The results of imputation analyses for cognitive ability were broadly consistent with those seen in the complete case analysis although the strength of the associations was attenuated. Conversely, the coefficients from the imputed analyses for educational attainment were much larger than for the complete case analysis, without overlapping confidence intervals, although the pattern of attenuation after adjustment for covariates was comparable. It is possible that this is due to the larger proportion of data being imputed or due to the larger sample size available for educational attainment compared to cognitive ability.

## Study Two

### Methods

#### Data sources

We conducted two-sample MR of two smoking phenotypes (smoking initiation and lifetime smoking) on cognitive ability and educational attainment. For the smoking initiation instrument, we used summary data for the 378 independent genome-wide significant SNPs identified by the GSCAN consortium GWAS (Liu et al., 2019). For lifetime smoking (combined smoking duration, cessation and heaviness), we used summary statistics reported by Wootton et al. (2019). The instrument consisted of 126 independent SNPs. For cognitive ability, we used a GWAS of fluid intelligence conducted in the UK Biobank by the Neale lab (http://www.nealelab.is/uk-biobank/). For the smoking initiation analysis, we used betas from the GSCAN GWAS with UK Biobank removed to avoid sample overlap. This was not possible for the lifetime smoking GWAS so this analysis was not conducted. For educational attainment, we used the second GWAS from the SSGAC consortium (Okbay et al., 2016) discovery sample. This GWAS was chosen rather than a more recently published GWAS (Lee et al., 2018) as there was no sample overlap between exposure and outcome samples (the more recent GWAS uses Biobank data, as does the smoking GWAS). Educational attainment was a harmonised measure defined as typical number of years of schooling to complete a given qualification.

#### Statistical analysis

We conducted four methods of Mendelian randomisation: inverse-variance weighted, MR Egger (Bowden, Davey Smith, & Burgess, 2015), weighted median (Bowden, Davey Smith, Haycock, & Burgess, 2016), weighted mode (Hartwig, Davey Smith, & Bowden, 2017). Each makes different assumptions about the presence of pleiotropy. A consistent direction of effect across all four methods provides the strongest evidence for a causal effect. We further calculated the MR Egger intercept, an indicator of directional pleiotropy (Bowden et al., 2015). Finally, Steiger filtering was used to confirm the hypothesised direction of effect (Hemani, Tilling, & Davey Smith, 2017). Here, each SNP is examined individually to ensure that it explains more variance in the exposure than the outcome. If a SNP explains more variance in the outcome, this could indicate that the outcome actually precedes the exposure on the causal pathway. Where this occurs, all analyses are re-run with these SNPs removed. All analyses were conducted using MR Base, a package for two-sample MR (Hemani et al., 2018) in R (R Core Team, 2013).

As a sensitivity analysis, multivariable MR (MVMR) was performed to investigate the independent effects of genetic liability to ADHD and smoking on cognitive ability and educational attainment (Burgess & Thompson, 2015; Sanderson, Davey Smith, Windmeijer, & Bowden, 2018). If genetic liability to smoking initiation includes elements of impulsivity or other related (e.g. risk-taking) traits, simultaneously modelling genetic liability to ADHD (as a marker of impulsivity) and smoking should help to disentangle this relationship. MVMR is an extension of MR that uses genetic variants associated with multiple exposures to simultaneously model the causal effect of each exposure on the outcome (Burgess & Thompson, 2015). These analyses were performed in the ALSPAC dataset from Study One.

Further sensitivity analyses using the ALSPAC dataset investigated associations between a standardised polygenic risk score for lifetime smoking and smoking initiation with IQ measured at age 8 using the WISC. This was to investigate the association between genetic variants associated with smoking and the outcome of interest prior to any smoking having occurred, as a negative control to test the assumption of MR that these genetic variants have no direct effect on general cognitive ability aside from via smoking. There was no appropriate educational attainment variable in ALSPAC at this age, therefore we were unable to do the same for this outcome. Polygenic risk scores were calculated using PLINK ‘score’ command. Genome-wide significant SNPs were identified in the previous GWAS of lifetime smoking (Wootton et al., 2019) and smoking initiation (Liu et al., 2019) and weighted by the effect sizes found in these GWAS. The WISC continuous total score was used as the outcome. We also conducted MVMR on this negative control outcome of general cognitive ability at age 8 years, including the ADHD SNPs mentioned above, to explore the independent effects of genetic liability for smoking and impulsivity in our negative control design.

### Results

#### Educational attainment (MR of summary data)

*Univariable MR.* Of the 126 SNPs associated with lifetime smoking (Wootton et al., 2019), all were available in the GWAS of educational attainment (Okbay et al., 2016) and the GWAS of cognitive ability (http://www.nealelab.is/uk-biobank/). Of the 378 SNPs associated with smoking initiation (Liu et al., 2019), 371 were available in the GWAS of educational attainment (Okbay et al., 2016) and 376 were available in the GWAS of cognitive ability (http://www.nealelab.is/uk-biobank/). For both smoking phenotypes, there was a consistent adverse effect of smoking on educational attainment across all four MR methods (see Table 4). The evidence was strongest for the effect of smoking initiation on educational attainment with genetic risk for smoking initiation associated with a 0.39 s.d. decrease in years of schooling (equating to 1.4 years of schooling) (95% CI −0.456 to −0.330, *p* = 1.35 × 10^−34^). Both MR Egger intercepts suggested weak evidence of directional pleiotropy. After Steiger filtering, 94% of SNPs were retained for the lifetime smoking analysis, and 75% of SNPs for the smoking initiation analysis (see Table 5), and the resultant analyses found strong evidence for an effect of smoking on educational attainment (e.g. for lifetime smoking, using 119 of the 126 original SNPs IVW MR −0.359, 95% CI −0.418 to −0.299, *p* = 1.81 × 10^−32^).

**Table 4.** Two-sample Mendelian randomisation analyses using summary statistics of the effect of smoking on educational attainment and cognitive ability

Exposure	Outcome	Method	*N* SNPs	*β* (95% CI)	*p* value
Smoking initiation (GSCAN)	Cognitive ability	Inverse-Variance Weighted	376	−0.817 (−1.038 to −0.595)	5.36 × 10^−13^
		MR Egger	376	−0.469 (−0.952 to 0.013)	0.057
		Weighted Median	376	−0.597 (−0.854 to −0.340)	5.22 × 10^−06^
		Weighted Mode	376	−0.469 (−1.274 to 0.336)	0.254
Smoking initiation (GSCAN)	Educational attainment	Inverse-Variance Weighted	371	−0.197 (−0.223 to −0.171)	1.78 × 10^−49^
		MR Egger	371	−0.145 (−0.254 to −0.035)	0.009
		Weighted Median	371	−0.159 (−0.185 to −0.132)	1.07 × 10^−31^
		Weighted Mode	371	−0.162 (−0.251 to −0.073)	4.15 × 10^−04^
Lifetime smoking	Educational attainment	Inverse-Variance Weighted	126	−0.393 (−0.456 to −0.330)	1.35 × 10^−34^
		MR Egger	126	−0.084 (−0.333 to 0.165)	0.509
		Weighted Median	126	−0.254 (−0.321 to −0.188)	4.84 × 10^−14^
		Weighted Mode	126	−0.163 (−0.334 to −0.009)	0.065

**Table 5.** Two-sample Mendelian randomisation analyses using summary statistics of the effect of smoking on educational attainment and cognitive ability after Steiger filtering

Exposure	Outcome	Method	*N* SNPs	*β* (95% CI)	*p* value
Smoking initiation (GSCAN)	Cognitive ability	Inverse-Variance Weighted	52/376 (14%)	0.042 (−0.293 to 0.377)	0.81
		MR Egger		0.249 (−0.749 to 1.248)	0.63
		Weighted Median		0.093 (−0.346 to 0.531)	0.68
		Weighted Mode		0.226 (−0.446 to 0.898)	0.51
Smoking initiation (GSCAN)	Educational attainment	Inverse-Variance Weighted	277/371 (75%)	−0.109 (−0.128 to −0.091)	1.85 × 10^−30^
		MR Egger		−0.149 (−0.224 to −0.075)	1.12 × 10^−04^
		Weighted Median		−0.129 (−0.156 to −0.103)	1.62 × 10^−21^
		Weighted Mode		−0.162 (−0.265 to −0.060)	0.002
Lifetime smoking	Educational attainment	Inverse-Variance Weighted	119/126 (94%)	−0.359 (−0.418 to −0.299)	1.81 × 10^−32^
		MR Egger		−0.092 (−0.329 to 0.144)	0.44
		Weighted Median		−0.252 (−0.315 to −0.189)	1.21 × 10^−15^
		Weighted Mode		−0.158 (−0.338 to −0.023)	0.09

*Multivariable MR.* When estimating the independent causal effects of genetic liability to ADHD and smoking initiation, there was strong evidence of a negative effect of genetic liability to ADHD on educational attainment (−0.064, 95% CI −0.088 to −0.041, *p* = 6.86 × 10^−8^). We also found strong evidence of a negative effect of genetic liability to smoking initiation on educational attainment (−0.288, 95% CI −0.375 to −0.20, *p* = 2.33 × 10^−10^). Similar effects were found when looking at ADHD and lifetime smoking, with strong evidence of a negative effect of genetic liability to both ADHD (−0.073, 95% CI −0.104 to −0.042, *p* = 2.41 × 10^−6^) and lifetime smoking (−0.279, 95% CI −0.362 to −0.197, *p* = 1.93 × 10^−11^) on educational attainment. These results are shown in Table 6. However, in both analyses, there was evidence of pleiotropy and instrument strength was weak (online Supplementary Table S3).

**Table 6.** Multivariable Mendelian randomisation analyses using summary statistics simultaneously modelling genetic liability to ADHD and smoking on educational attainment or cognitive ability

Exposure	Outcome	*N* SNPs	*β* (95% CI)	*p* value
Smoking initiation (GSCAN)	Cognitive ability	322	−0.405 (−0.721 to −0.089)	0.012
ADHD			−0.171 (−0.257 to −0.085)	5.63 × 10^−5^
Smoking initiation (GSCAN)	Educational attainment	320	−0.288 (−0.375 to −0.200)	2.33 × 10^−10^
ADHD			−0.064 (−0.088 to −0.041)	6.83 × 10^−8^
Lifetime smoking	Educational attainment	114	−0.279 (−0.362 to −0.197)	4.06 × 10^−10^
ADHD			−0.073 (−0.104 to −0.042)	3.41 × 10^−6^

#### General cognitive ability

Analyses exploring the association between smoking and general cognitive ability were inconsistent, and sensitivity analyses indicated potential pleiotropic effects of the smoking SNPs. We found evidence of association of these with general cognitive ability at age 8 in ALSPAC, prior to smoking occurring, even after taking impulsive traits in to account (see online Supplementary Results and Supplementary Table S6).

### Discussion

These results provide some evidence for a causal effect of smoking on education. The results for cognitive ability are more mixed, but may indicate common genetic influence on both cognition and smoking. Interestingly, there was stronger evidence for pleiotropy in the analysis of lifetime smoking on educational attainment, compared with the analysis of smoking initiation on educational attainment. Given that educational attainment is largely realised by early adulthood, smoking initiation may be the more appropriate exposure to examine (unlike for most outcomes, where the effects of smoking develop over a lifetime of exposure). We have also previously shown that educational attainment impacts on smoking (Davies et al., 2018; Gage et al., 2018; Sanderson et al., 2019), with these effects likely to continue to play out over the lifetime (e.g. via effects on the heaviness of smoking and smoking cessation). Interestingly, multivariable MR analyses suggest that there is no independent effect of general cognitive ability on smoking when the effect of educational attainment is taken into account (Sanderson et al., 2019).

Taken together, these results and our previous findings indicate the possibility of a complex, bidirectional causal relationship between smoking and educational attainment, which we explore further below, in the context of our findings in Study One. Evidence for the causal effect of smoking on cognition is more mixed. This is perhaps to be expected, as cognition is less susceptible to the impact of the environment than educational attainment is likely to be (Ritchie & Tucker-Drob, 2018). It is also important to bear in mind that MR analyses between lifetime smoking and cognitive ability were not possible due to the use of UK Biobank data in both the exposure and outcome GWAS. The implementation of Steiger filtering and subsequent removal of 86% of the 376 initial SNPs may indicate evidence of reverse causality or a common biological cause, although power is reduced for these analyses due to the removal of identified SNPs. Critically, the results of our negative control analyses, which indicate that the genetic instruments for smoking are associated with general cognitive ability before individuals have begun smoking, suggests there may be direct effects of these variants on cognition. One possibility is that these variants capture a common mechanism (e.g. trait impulsivity) that has effects on both smoking and cognition. It is plausible that impulsivity during adolescence might impact on both educational attainment and the likelihood of smoking directly. We explored this in our MVMR analysis, using variants associated with ADHD as a proxy for trait impulsivity. Whilst we found some evidence for independent effects of ADHD variants and smoking variants on the outcomes, there was also evidence of pleiotropy, and the instruments themselves were weak. We cannot exclude the possibility that variants associated with smoking initiation reflect a common mechanism (e.g. a broad impulsivity phenotype not adequately captured using ADHD), are predictive of maternal smoking during pregnancy, or that smoking could be on the causal pathway between trait impulsivity and educational attainment or cognition. It will be important to address these questions in future research.

## General discussion

Our study provides observational evidence that smoking is associated with reduced general cognitive ability and educational attainment (Study One), and MR evidence that smoking may have a causal effect on educational attainment (Study Two). This supports the importance of preventive interventions in schools to reduce the uptake of smoking in young people. The triangulation of results across different methods, each with their own strengths, limitations and sources of bias, is an important strength of our approach.

However, there are a number of limitations to this study that should be considered. First, smoking was based on self-report as there was no objective measure available in either our observational or MR analyses. Second, in our observational analyses, the WASI was not used in its entirety and therefore the estimated score was less reliable than a full IQ score (Mokrysz et al., 2016). Third, our observational analyses are cross-sectional, meaning reverse causality cannot be ruled out, although we attempt to minimise any impact by adjusting for earlier cognitive ability or educational attainment. Fourth, we cannot exclude the possibility that a common risk factor is operating that influences both smoking and lower cognitive abilities and educational attainment, for example, impulsivity. Fifth, whilst MR can in some circumstances provide more precise estimates of causal effects, this is not advisable when cigarette smoking is the exposure (Taylor et al., 2014). Sixth, genetic variants exert their effects across the lifespan, but we were interested in an exposure during a particular developmental period. We therefore triangulated results of conventional observational analyses in a dataset that provided the temporal granularity to focus on this period with those of MR analyses, which in principle support stronger causal inference (Holmes, Ala-Korpela, & Davey Smith, 2017).

MR should in principle allow us to assess the potential causal pathway between smoking and education without confounding from socio-demographic factors if the assumptions of MR have been met. However, if individuals who smoke are more likely to reproduce with other individuals who smoke, as some studies have indicated (Agrawal et al., 2006), the assumptions required by MR may not hold. Given the social patterning of smoking behaviour, cigarette use might be a marker for many aspects of deprivation that would likely impact on years of education (for example, the need to leave school to begin employment), an interpretation that cannot be ruled out by these data.

Relatedly, it may be the case that the phenotypes used are capturing different phenotypes to those intended. Asking an individual whether they have ever tried smoking, for example, may capture traits such as impulsivity or risk-taking, rather than smoking itself (although in the GSCAN GWAS participants had to have smoked 100 cigarettes in their lifetime or been a regular smoker at some point). The results of our negative control analyses in ALSPAC provide some evidence for this possibility. Triangulation of results using Steiger filtering and negative control analyses indicated that the genetic variants associated with smoking initiation have pleiotropic effects, and MVMR analyses using genetic variants associated with ADHD as a proxy for trait impulsivity was not sufficient to account for this. This highlights the importance of conducting and triangulating sensitivity analyses to test MR assumptions.

Therefore, whilst our results suggest that smoking may have a causal effect on educational attainment above and beyond the effects of impulsivity (again using ADHD as a proxy for impulsivity), a more complex pattern is possible. Smoking may lie on the causal pathway between impulsive traits and cognition and education, or the genetic variants associated with smoking initiation may be pleiotropic, and directly influence cognition, which in turn influences educational outcomes (meaning that smoking does not play a causal role). This warrants further research, as it is not possible to resolve this uncertainty in our data. Crucially, there is evidence from other studies using different designs that do not have these limitations, which suggests that smoking might causally impact on educational attainment. This includes instrumental variable analyses in different cultural settings (Zhao, Konishi, & Glewwe, 2010), allowing for further triangulation of results.

It has long been suggested that nicotine may be a cognitive enhancer, which is apparently paradoxical given our results. The mechanisms by which cigarette use might impact on educational attainment are harder to elucidate. There is some evidence to suggest that smoking may lead to adverse mental health outcomes, or externalising behaviours (Gage et al., 2017). It is possible that these, in turn, could influence educational outcomes and/or cognitive ability; alternatively, effects may be socially-mediated (for example, via peer groups where smoking is common, leading in turn to other activities that impact on educational outcomes). However, our data cannot interrogate these possibilities directly.

We used two different methodologies to investigate the link between smoking and education and cognitive ability. Results from both studies suggest that smoking may affect educational attainment as early in life as adolescence. While both methods used have limitations, they have different limitations, and therefore the consistency across both methods – together with evidence from other studies – provides greater confidence in our results. Future research should also explore the potentially complex causal pathways (and potentially non-causal associations) between cognitive ability, educational outcomes, smoking, and other phenotypes such as a general tendency to risk-taking or impulsivity. If smoking does have a causal effect on educational outcomes, it will be important to ascertain the mechanisms that underpin this. This, in turn, could inform early interventions aimed at preventing smoking uptake, for example within the school environment, as these could support higher educational attainment and, ultimately, reduce the health and social inequality that derives from both smoking and low education.

## Supplementary material

For supplementary material accompanying this paper visit https://doi.org/10.1017/S0033291720003402. click here to view supplementary material
