# Supplementary material for: Does smoking cause lower educational attainment and general cognitive ability? Triangulation of causal evidence using multiple study designs
Source: Psychol Med. 2020 Oct 7;52(8):1578–86. doi: 10.1017/S0033291720003402 (PMC9226381; doi:10.1017/S0033291720003402)
Supplement: Supplementary file 1 [file S0033291720003402sup001.docx]

**Does Smoking Cause Lower Educational Attainment and General Cognitive Ability?**

**Triangulation of causal evidence using multiple study designs**

**Supplementary Results**

*General Cognitive Ability.*

*Univariable MR.* Using published summary-level data, there was some evidence for an adverse effect of smoking initiation on cognitive ability (IVW MR -0.82, 95% CI -1.04, -0.60, P = 5.36 x 10^-13^), and the direction of effect was consistent across all methods (see Table 4 in the main article). Steiger filtering indicated that 86% of the SNPs for smoking initiation explained more variance in cognitive ability than smoking phenotypes (see Table 5 in the main article). After Steiger filtering, the effects of smoking initiation on cognitive ability attenuated to the null (IVW MR 0.04, 95% CI -0.29 to 0.38, P = 0.81).

*Multivariable MR.* When estimating the causal effect of both genetic liability to ADHD and smoking initiation, there was strong evidence of a negative effect of genetic liability to ADHD on cognitive ability (-0.171, 95% CI -0.26 to -0.09, P = 9.14 x 10^-5^), independently of smoking initiation. There was also some evidence of a negative effect of genetic liability to smoking initiation on cognitive ability (-0.405, 95% CI -0.72 to -0.09, P = 0.012), independently of ADHD. However, there was some evidence of pleiotropy within these instruments and instrument strength was weak. As such these estimates should be interpreted with caution. These results are shown in Table 6 in the main article.

*Sensitivity analyses.* Using ALSPAC data, we found evidence of associations between polygenic risk scores for both smoking initiation and lifetime, with general cognitive ability measured at age 8 years. This was true in the whole sample (N=5,300), and when restricted to individuals who reported never having tried a cigarette (N=4,650). These results are shown in Supplementary Table S4. MVMR examining smoking and ADHD on general cognitive ability at age 15 was also run in the ALSPAC sample, although this was very underpowered (see Supplementary Table S5). The negative control MVMR examining smoking and ADHD on general cognitive ability at age 8 was also underpowered, but indicated that SNPs associated with smoking initiation and lifetime smoking were associated with general cognitive ability at age 8, prior to any smoking behaviour in the sample, even after accounting for impulsivity as proxied by ADHD (Supplementary Table S6).

**Supplementary Tables**

**Supplementary Table S1: Linear regression of cigarette use heaviness at age 15 and general cognitive ability at age 15 in ALSPAC using imputed data (100 imputations), before and after adjustment for potential confounders (N=4,954)*.**

| Model | Beta coefficient | 95% CI | P value |
| --- | --- | --- | --- |
| 1 | -1.166 | -1.429, -0.923 | <0.001 |
| 2 | -0.710 | -0.927, -0.493 | <0.001 |
| 3 | -0.583 | -0.799, -0.367 | <0.001 |
| 4 | -0.553 | -0.775, -0.332 | <0.001 |
| 5a | -0.527 | -0.823, -0.231 | <0.001 |
| 5b | -0.533 | -0.800, -0.266 | <0.001 |
| 5c | -0.500 | -0.748, -0.253 | <0.001 |
| 6 | -0.488 | -0.805, -0.171 | 0.003 |

Model 1 – Standardized general cognitive ability score at age 15 by unit increase of 5-level smoking heaviness category at age 15; Model 2 – as model 1 with additional adjustment for general cognitive ability at age 8; Model 3 – as model 2 with additional adjustment for pre-birth confounders (gender, maternal education, maternal smoking, alcohol and cannabis use during pregnancy, maternal depression); Model 4 – as model 3 with additional adjustment for childhood confounders (truancy, hyperactivity, conduct problems, psychotic-like experiences, depression); Model 5a – as model 4 with additional adjustment for cannabis use at age 15; Model 5b – as model 4 with additional adjustment for alcohol use at age 15; Model 5c – as model 4 with additional adjustment for illicit drug use at age 15; Model 6 – as model 4 with additional adjustment for cannabis, alcohol and other illicit drug use at age 15. * The imputed interaction model allowed us to account for participants who had maternal report of head injury resulting in unconsciousness, without exclusions.

**Supplementary Table S2: Linear regression of cigarette use heaviness at age 15 and educational attainment score at age 16 in ALSPAC using imputed data (100 imputations), before and after adjustment for potential confounders (N=12,004)*.**

| Model | Beta coefficient | 95% CI | P value |
| --- | --- | --- | --- |
| 1 | -4.695 | -5.052, -4.337 | <0.001 |
| 2 | -3.093 | -3.380, -2.807 | <0.001 |
| 3 | -3.047 | -3.330, -2.764 | <0.001 |
| 4 | -2.587 | -2.895, -2.279 | <0.001 |
| 5a | -2.019 | -2.429, -1.608 | <0.001 |
| 5b | -2.437 | -2.797, -2.078 | <0.001 |
| 5c | -2.138 | -2.489, -1.787 | <0.001 |
| 6 | -1.865 | -2.292, -1.438 | <0.001 |

Model 1 – Educational performance score (as % of total possible) at age 16 by unit increase of 5-level smoking heaviness category at age 15; Model 2 – as model 1 with additional adjustment for educational attainment at age 11; Model 3 – as model 2 with additional adjustment for pre-birth confounders (gender, maternal education, maternal smoking, alcohol and cannabis use during pregnancy, maternal depression); Model 4 – as model 3 with additional adjustment for childhood confounders (truancy, hyperactivity, conduct problems, psychotic-like experiences, depression); Model 5a – as model 4 with additional adjustment for cannabis use at age 15; Model 5b – as model 4 with additional adjustment for alcohol use at age 15; Model 5c – as model 4 with additional adjustment for illicit drug use at age 15; Model 6 – as model 4 with additional adjustment for cannabis, alcohol and other illicit drug use at age 15. * The imputed interaction model allowed us to account for participants who had maternal report of head injury resulting in unconsciousness, without exclusions.

**Supplementary Table S3: Multivariable Mendelian Randomization (MVMR) heterogeneity measures (using summary statistics).**

| Outcome |  |  | Q | P-value |
| --- | --- | --- | --- | --- |
| Cognitive ability | Pleiotropy |  | 947.7 | 2.23x10^-63^ |
|  | Instrument strength | ADHD | 1233.5 | 2.18x10^-105^ |
|  |  | Smoking initiation | 1091.0 | 1.55x10^-84^ |
| Educational attainment | Pleiotropy |  | 780.7 | 1.86x10^-40^ |
|  | Instrument strength | ADHD | 1224.0 | 4.71x10^-106^ |
|  |  | Smoking initiation | 1114.5 | 1.04x10^-88^ |
|  | Pleiotropy |  | 266.7 | 1.51x10^-14^ |
|  | Instrument strength | ADHD | 659.3 | 4.90x10^-78^ |
|  |  | Lifetime smoking | 546.7 | 4.38x10^-58^ |

**Supplementary Table S4. Associations between standardised polygenic risk scores for lifetime smoking (LS) and smoking initiation (SI) with IQ (measured as total score on the WISC) at age 8 in the ALSPAC offspring**.

|  |  | **Beta** | **95% CI** | **P-value** | **N** |
| --- | --- | --- | --- | --- | --- |
| Whole sample | LS | -0.934 | -1.37, -0.50 | 2.8x10^-5^ | 5,300 |
|  | SI | -0.749 | -1.17, -0.30 | 9.0x10^-4^ | 5,300 |
| Restricted to individuals who report never trying a cigarette | LS | -0.893 | -1.34, -0.44 | 1.0x10^-4^ | 4,950 |
|  | SI | -0.785 | -1.24, -0.33 | 6.3x10^-4^ | 4,950 |

Analyses are adjusted for sex, age, and first 5 principal components.

**Supplementary Table S5: Multivariable Mendelian Randomization (MVMR) analyses using the ALSPAC dataset simultaneously modelling genetic liability to ADHD and smoking initiation on cognitive ability at age 15, using two genetic instruments for smoking.**

|  | Model | Effect estimate | 95% CI | P-value | F-statistic | N |
| --- | --- | --- | --- | --- | --- | --- |
| Analysis using genetic variants for smoking initiation to proxy for ‘ever smoked’ at age 15 | | | | | | |
| ADHD (age 15) | Observational* | -7.45 | -9.60,-5.30 | 1.37x10^-11^ | - | 3,568 |
|  | Multivariable observational^ | -7.20 | -9.38,-5.02 | 1.04x10^-10^ | - | 3,491 |
|  | Mendelian randomization | -3.06 | -34.06, 27.94 | 0.847 | 2.00 | 3,568 |
|  | Multivariable Mendelian randomization | -1.17 | -7.62,5.28 | 0.722 | 0.46 | 3,491 |
|  |  |  |  |  |  |  |
| Ever smoked (age 15) | Observational* | -4.22 | -5.11, -3.33 | 1.98x10^-20^ | - | 3,867 |
|  | Multivariable observational* | -4.20 | -5.12,-3.27 | 9.97x10^-19^ | - | 3,491 |
|  | Mendelian randomization | -4.61 | -7.26,-1.97 | 0.001 | 1.50 | 3,867 |
|  | Multivariable Mendelian randomization | -4.42 | -7.06,-1.77 | 0.001 | 1.51 | 3,491 |
| Analysis using genetic variants for lifetime smoking to proxy for ‘lifetime smoking’ at age 15 | | | | | | |
| ADHD (age 15) | Mendelian randomization | -3.06 | -34.06, 27.94 | 0.847 | 2.00 | 3,568 |
|  | Multivariable MR | -4.74 | -16.36,6.89 | 0.424 | 0.92 | 3,491 |
|  |  |  |  |  |  |  |
| Ever smoked (age 15) | Mendelian randomization | -7.60 | -11.80,-3.41 | 1.9x10^-4^ | 1.39 | 3,867 |
|  | Multivariable MR | -8.55 | -12.73,-4.36 | 3.17x10^-5^ | 1.26 | 3,491 |

* Adjusted for sex, age and PCs

^ Additionally adjusted for ADHD/smoking

**Supplementary Table S6: Multivariable Mendelian Randomization (MVMR) analyses using the ALSPAC dataset simultaneously modelling genetic liability to ADHD and smoking initiation on cognitive ability at age 8, using two genetic instruments for smoking.**

|  | Model | Effect estimate | 95% CI | P-value | F-statistic | N |
| --- | --- | --- | --- | --- | --- | --- |
| Analysis using genetic variants for smoking initiation to proxy for ‘ever smoked’ at age 15 | | | | | | |
| ADHD (age 15) | Observational* | -10.52 | -13.4, -7.6 | <0.001 | - | 3,218 |
|  | Multivariable observational^ | -9.76 | -12.7, -6.8 | <0.001 | - | 3,160 |
|  | Mendelian randomization | -2.84 | -37.5, 31.8 | 0.872 | 2.52 | 3,218 |
|  | Multivariable Mendelian randomization | -5.33 | -13.7, 3.0 | 0.210 | 0.39 | 3,160 |
|  |  |  |  |  |  |  |
| Ever smoked (age 15) | Observational* | -3.44 | -4.6, -2.3 | <0.001 | - | 3,518 |
|  | Multivariable observational* | -3.18 | -4.4, -2.0 | <0.001 | - | 3,160 |
|  | Mendelian randomization | -5.90 | -9.4, -2.4 | 0.001 | 1.36 | 3,518 |
|  | Multivariable Mendelian randomization | -4.55 | -8.0, -1.1 | 0.010 | 1.37 | 3,160 |
| Analysis using genetic variants for lifetime smoking to proxy for ‘lifetime smoking’ at age 15 | | | | | | |
| ADHD (age 15) | Mendelian randomization | -2.84 | -37.5, 31.8 | 0.872 | 2.52 | 3,218 |
|  | Multivariable Mendelian randomization | -16.10 | -30.8, -1.4 | 0.031 | 0.84 | 3,160 |
|  |  |  |  |  |  |  |
| Ever smoked (age 15) | Mendelian randomization | -8.31 | -13.8, -2.8 | 0.003 | 1.29 | 3,518 |
|  | Multivariable Mendelian randomization | -7.83 | -13.4, -2.2 | 0.006 | 1.17 | 3,160 |

* Adjusted for sex, age and PCs

^ Additionally adjusted for ADHD/smoking
